# Selenium’s Emergence from the Pool of Potentially Essential Trace Elements

**DOI:** 10.1007/s12011-026-05042-4

**Published:** 2026-03-23

**Authors:** Roger A. Sunde

**Affiliations:** Department of Nutritional Sciences, University of Wisconsin, 1415 Linden Dr, WI 53706 Madison, USA

## Abstract

Animal research showed that selenium (Se) was a dietary trace nutrient that would protect against disease, and that it often interacted with vitamin E in this protection. Studies reporting that Se was toxic and could cause cancer in animal models, however, prevented direct Se supplementation of animal feeds, and prevented recommendations for human diets. The key for Se’s emergence from the pool of other potentially essential trace elements was the discovery by John Rotruck and William Hoekstra of a biochemical role for Se as a cofactor in glutathione peroxidase (Gpx). Gpx activity falls in Se deficiency and rises to a plateau with Se supplementation, demonstrating that Gpx was an effective biomarker for Se deficiency, and supported dietary recommendations. There remain 8 or so potentially essential trace elements for higher animals that have been shown to result in abnormalities when lacking in the diet. It is very likely that future research will discover functional roles for the elements in humans as these elements are recognized by other living organisms for specific transport or incorporation into proteins or metalospecies. The identity of these processes in higher animals, and the challenge of finding good biomarkers for excess nutrient intake will provide ample opportunity for future trace element research.

## Introduction

Dr. Forrest H. Neilsen has been a leader and champion of ultra trace element research since the 1960’s. He was a graduate student of my father, Milton Sunde, and my future Ph.D. mentor, William Hoekstra, at the University of Wisconsin (UW). He was a character even back then, so I remember him. “Frosty” was a cheerleader and someone that I talked with beginning when we attended the 2nd Trace Element Metabolism in Animals meeting in 1973 – he is one of my trace element heroes.

At UW, Frosty used chickens as an experimental model studying the role of zinc, and they published a series of four papers on zinc in poultry and its interaction with other dietary nutrients, agents, and chelators [1–4]. From there, he expanded this approach to study possible requirements for nickel in chicks while at Fitzsimons General Hospital in Denver, Colorado [5] and later in rats at The USDA in Grand Forks, North Dakota [6]. In these and later studies, Frosty adapted, developed, and refined tools using specialized trace-element deficient diets, plastic cages, mineral-free water, and even filtered air [7]. Frosty’s approaches became the standard for ultra-trace element research, and under his leadership the USDA Human Nutrition Laboratory in Grand Forks became the mecca for studying potentially essential trace elements for humans as well as animals [8]. And *Biological Trace Element Research* under his editorship was a centerpiece for those of us in the newer trace element research community.

To set the stage, the US Food and Nutrition Board in1968 (7th edition) listed Recommended Dietary Allowances for 6 elements (Ca, P, Cu, I, Fe, Mg) with discussion only for 6 additional trace elements (Cr, Co, Mn, Mo, Se, Zn) [9, 10]. In that era, researchers investigating trace elements employed a more universal set of methods regardless of target trace element, and met in international as well as national meetings that consisted of small groups of perhaps 100 or so scientists and covered the full range of elements under study. In addition to specialized diets and cages, these studies often followed more than one element, and looked for interactions or used radioactive tracers to observe signs of nutrient deficiency. Many of these efforts over the past five decades are summarized in the accompanying reports in our tributes to Dr. Nielsen.

## Trace Element Essentiality

There are a number of solid and recent reviews of Se nutrition, biochemistry, genomics, and molecular biology [11–15], but my purpose here is to discuss the emergence of Se from the pool of potentially essential trace elements. My current list of trace essential and potentially essential trace elements for higher animals is shown in Table 1. I’ll leave the detailed discussion of where these elements lie to another venue.

**Table 1:** Author’s classification of mineral elements in higher animals

Essential macro elements	Essential trace elements	Likely/Potential trace elements	Doubtful essential trace elements
Sodium	Iron	Nickel	Chromium
Potassium	Zinc	Boron	
Chloride	Copper	Vanadium	
Calcium	Selenium	Silicon	
Magnesium	Iodine	Tin	
Phosphorus	Fluoride	Arsenic	
Sulfur	Manganese	Tungsten	
(Oxygen)	Molybdenum	Cadmium	
	Cobalt	Lithium	

Essential classification requires a known biochemical role and biomarker in animals and other vertebrates. Likely/potential classification indicates occurrence of abnormalities in vertebrates reported, and biochemical roles observed in organisms at other phylogenetic levels (updated from ref [16])

In 1967, Cotzias [17] concisely framed six criteria for mineral essentiality. A trace element is essential if: (i) it is present in all healthy tissues of all living things; (ii) its concentration from one animal to the next is fairly constant; (iii) its withdrawal from the body induces reproducibly the same physiological and structural abnormalities regardless of species considered; (iv) its addition either reverses or prevents these abnormalities; (v) the abnormalities induced by deficiency are accompanied by pertinent, specific biochemical changes; (vi) these biochemical changes can be prevented or cured when the deficiency is prevented or cured. The listed essential trace elements meet criteria v and vi. Below I summarize how Se met these criteria.

## Signs of Se Essentiality

Se was first recognized in the 1930s as a toxic element based on studies in animals [18], and this was further supported by a report that dietary Se caused cancer in rats [19] (now concluded to be in error [20]). In contrast, epidemiology studies observed that Se status was inversely correlated with cancer, suggesting that low Se status may predispose individuals to cancer [21].

These views were superseded by the discovery that Se was the third factor, in addition to vitamin E and sulfur amino acids, that would prevent dietary liver necrosis in rats [22]. Diets low in Se resulted in this condition, and addition of Se prevented or reversed the disease. This was followed by demonstration that Se would prevent abnormalities in chickens fed a low Se/vitamin E diet and that Se supplementation would prevent or reverse the condition [23]. There followed a series of discoveries that domestic animal diseases were associated with low Se intake, and that Se supplementation would prevent the condition [24]. In the majority of cases, Se or vitamin E supplementation was preventative, but Se alone was necessary to prevent pancreatic atrophy [25]. Specific biochemical changes associated with these conditions, however, remained elusive. Even though Se supplementation was known to prevent domestic animal diseases, the reports that Se was toxic at some level, and that Se may promote cancer prevented addition of Se to livestock diets. Interestingly, naturally high Se wheat grown in the Dakotas (due to high soil Se) was highly sought by animal feed manufacturers to raise measured Se content without supplementation [26].

## Biochemical Roles for Se

A number of research groups thus began searching for a biochemical role for Se. As outlined by William Hoekstra in 1975 [27], his graduate student John Rotruck began a series of studies focused on red cells from rats fed diets low in Se and vitamin E. The key observation made by John Rotruck from the literature was that the glutathione (GSH) levels were reported to be elevated in RBCs from Se-deficient rats, indicating an inability to use vs. synthesize GSH, leading him to focus on glutathione peroxidase (Gpx). Over the summer in 1971, he adapted an assay for Gpx and found that Gpx activity was low in RBCs from Se-deficient rats as compared to Se supplemented rats [28–32]. Studies in the Hoekstra laboratory measured tissue Se concentrations in these animals; purified Gpx from ovine blood, demonstrated coelution of Gpx activity and Se in purified fractions, and found 4 (measured 3.84) Se atoms per tetramer of purified Gpx [33]. This clearly established a biochemical role for Se in higher animals. While the report that Gpx was a selenoenzyme was published in January 1973, the series of preceding published articles and abstracts for this work [28–32], including manuscript submission and acceptance dates, clearly document that John Rotruck and William Hoekstra were the ones who discovered that Gpx was a selenoenzyme. This series of studies also illustrates the process of exploring a ”crack” – a perhaps unexpected observation – that leads to new discovery.

**Fig. 1:**
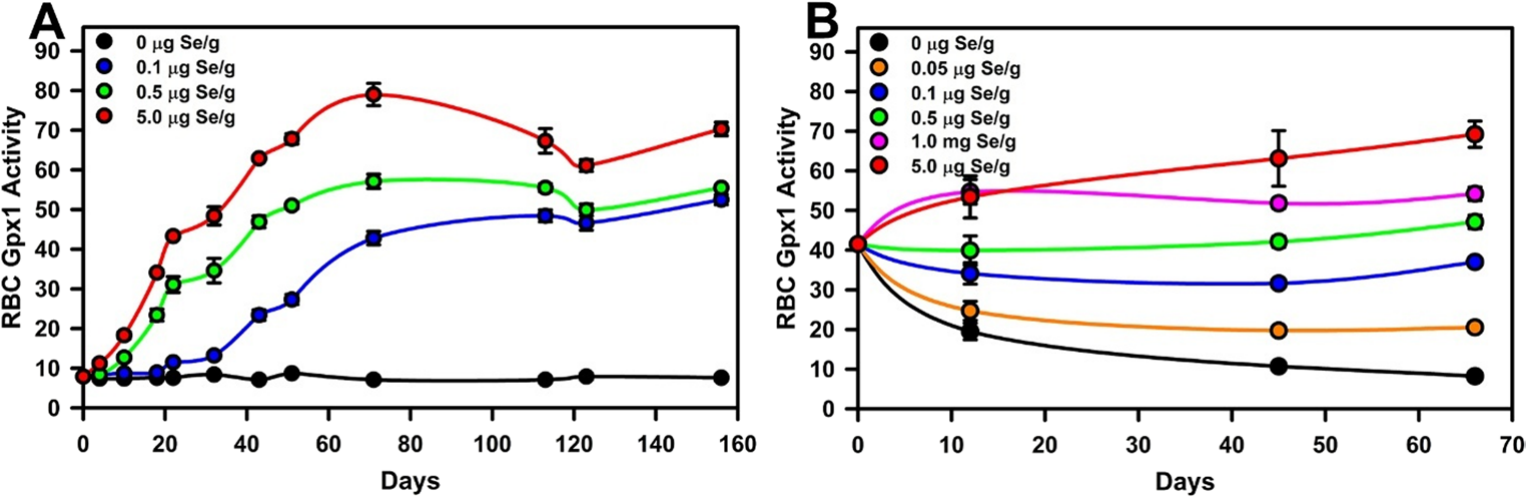
Effect of Se supplementation on Gpx activity in red cells (RBC). (**A**) Adult Se-deficient rats re-supplemented with Se. (**B**) Weanling rats supplemented with Se. Se provided as Na_2_SeO_3_. Data from ref. [34] replotted

Prof. Hoekstra was my biochemistry major advisor, and when I returned to UW in the fall of 1971, I was tasked with mixing diets and feeding rats torula-yeast diets containing adequate vitamin E but low in Se or supplemented with graded levels of sodium selenite, and with feeding adult Se-deficient rats with graded Se supplementation [34]. RBC Gpx activity fell to near zero in Se-deficient rats, and was restored with Se supplementation to Se-adequate levels (Fig. 1A). Feeding graded levels of Se to weanling rats showed that Gpx activity fell in rats fed the Se-deficient diet, and then reached a plateau, showing that Se was no longer the factor limiting accumulation of Gpx activity (Fig. 1B). These studies demonstrated that Gpx was an excellent biomarker for assessing Se status and Se requirements.

**Fig. 2:**
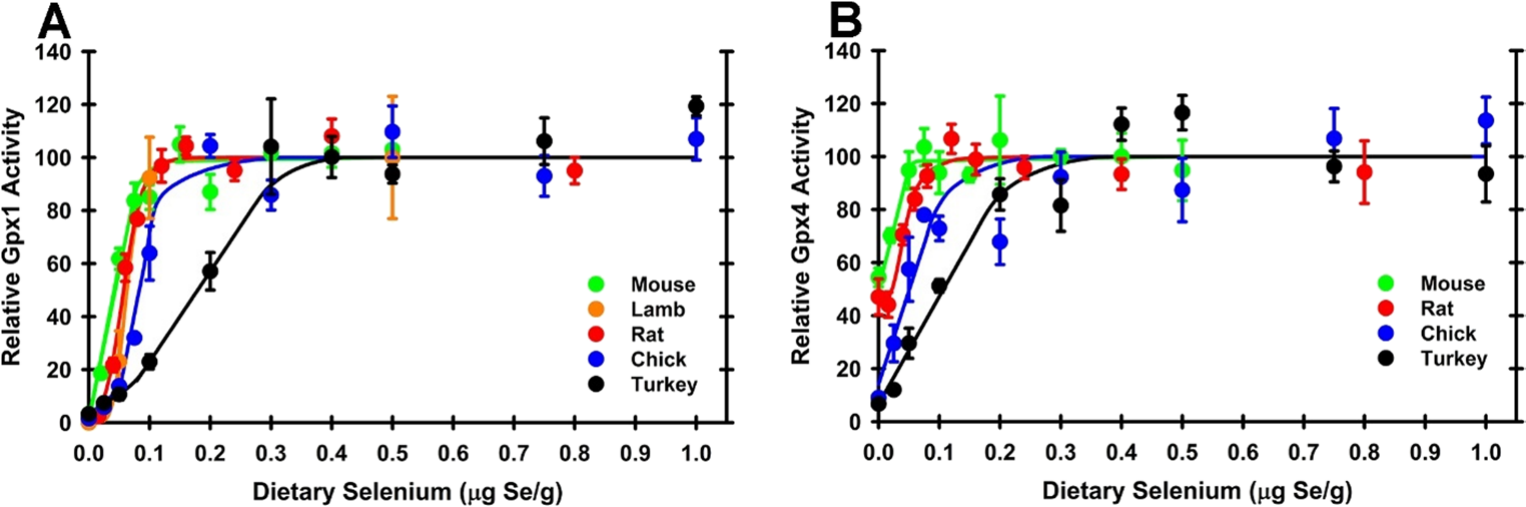
Effect of dietary Se on Gpx activity in liver in animals supplemented with graded levels of Se. (**A**) Relative Gpx1 activity in 5 species. (**B**) Relative phospholipid hydroperoxide Gpx (Gpx4) activity in 4 species. Figures from ref. [13]

Subsequent studies in sheep, pigs, chickens, and cattle using Gpx as a Se status biomarker demonstrated that Se supplementation would prevent associated livestock disease, and was not toxic or cancer promoting when supplemented up 2 µg Se/g diet, permitting animal fed manufacturers to add Se directly to animal diets [24, 26]. Figure 2A illustrates the usefulness of Gpx1 activity as a biomarker across the full range from 0.005 to 5 µg Se/g diet, falling dramatically in deficiency, rising to a plateau with increasing Se supplementation to allow solid assessment of adequate Se intake, and with minimal change above the requirement. Note the distinctly higher Se requirement for Gpx1 activity for turkeys and to a lesser extent chickens, illustrating species differences. Also shown is the activity of liver Gpx4 in these same animals, with rodent liver Gpx4 activity only falling about 50% in Se deficiency (Fig. 2B). Notable here is that turkeys have 10 times the level of liver Gpx4 activity as compared to rodents, vs. only 1/6th the level for Gpx1 activity [13, 35]; underlying these differences are differential effects on regulation of the corresponding mRNAs by Se status [13]. These examples illustrate the effectiveness of a good biomarker for nutrient status, as well as warning that there are likely to be differences when extrapolating to different species or tissues.

Se research today has clearly joined (rejoined? ) biology/molecular biology research. We know that Se is a cofactor for 24–25 mammalian selenoproteins [12]. Deletion of some is fetally lethal or leads to serious defects. Se incorporation into these proteins occurs at the UGA codon, normally a stop codon, and this requires several unique and novel enzymes or RNA species [11, 12]. Furthermore, the biological roles for these selenoproteins are diverse. The result is that Se research has continuously been infused by exciting researchers from other areas bringing novel and new techniques and approaches. This profile is thus dramatically different from the profile of trace element research in the 1960s, and we all benefit from this.

## Se-Vitamin E Interactions

The initial observations, that vitamin E or Se alone would prevent animal disease signs, established that these nutrients were involved in preventing lipid peroxidation and reactive oxygen. The specific roles in this prevention, however, remained largely elusive. The one clear discovery is that the selenoenzyme Gpx4, vitamin E, and cysteine as a component of glutathione, together function to limit ferroptosis [36]. This clearly provides details in the role of vitamin E as well biochemical basis for this Se-vitamin E interaction. And it offers a crack to be explored to further understand the molecular role of vitamin E.

## Essentiality of Other Trace Elements

Demonstration of a biochemical role in biology remains critical for establishing essentiality. This was Frosty’s quest throughout his career, and he made and championed research to find animal models meeting Cotzias’ criteria i-iv [37]. My listing in Table 1 as Likely/Probable indicates that while a biochemical role has not been identified in animals, metal-dependent enzymes or biological functions have been identified in plants and/or microorganisms [16, 38, 39].

For instance, Dr. Nielsen and others reported signs of deficiency for nickel [5, 6], including decreased growth in second-generation rats [40]; at least eight enzymes with Ni cofactors have been found in plants and micro-organisms [41]. While clear-cut demonstrations of boron essentiality in humans has not been shown [42], there is convincing evidence in fish for a boron requirement for growth [43]; in plants, boron supplementation is needed for citrus, and clear-cut gene deletion studies have demonstrated that boron is needed for pollen tube growth in Arabidopsis [44]. In addition, a series of boron-specific transporters have been identified in plants, and homolog genes for these transporters in higher animals further support future demonstration of boron essentiality [45]. Results showing increased amyloid-β plaques in an Alzheimer’s disease mouse model using a Li-depleted diet [46] has shifted Li into the potential essential category for this author.

Similar bodies of evidence for potential roles for V, Si, Sn, As, and Cd have been reviewed [39]. Defined roles in biology for Si accumulation and transporters in plants [47], and tungsten-specific cofactors, distinct from the molybdenum cofactor, are found in bacteria [48]. These examples show that biology has evolved to recognize and value additional trace elements beyond those currently identified as essential in humans.

These and other reports are emerging clues that may help find biochemical roles for some of the remaining potentially essential trace element. Regardless of whether it needs to be supplemented in the diet, it should remain that identification of a catalytic or participatory role for a trace element in a critical biochemical pathway is the gold standard for essentiality. This is what promoted Se out of the pool of potentially essential trace elements.

In Table 1, Cr remains in the Doubtful category as there is no confirmed evidence for a biological role for Cr. In the quest for establishing chromium as an essential nutrient, an addition criterion was suggested: “An element is considered essential if its deficiency consistently results in impairment of function from optimal to suboptimal.” [49]. This present author, however, asserts that this approach and the use of epidemiology studies, including meta-analysis, is at best discovery science exploring potential essentiality, with the danger of leading to overstate nutrient requirements and recommendations to the public.

## Biomarkers of Nutrient Toxicity

The story of essential trace elements is not over. The makers of food supplements and animal feed manufacturers keep advocating and providing higher levels of nutrients including trace elements in their products. What is missing is the identification of good biomarkers for toxic and high nutrient status. Trace element concentrations themselves are a start, but there must be homeostatic mechanisms that detect when increasing nutrient status ceases to be advantageous, and which activate processes to modulate status. Down-regulation of uptake metal transporters, up-regulation of metal export or excretion, or increased synthesis of metalospecies for excretion or accumulation, are some of the ways that this could occur. For Se, methylated Se species and selenosugar metabolism [50, 51] have potential as high Se status biomarkers. This is an area for future exploration in our quest to fully understand trace element essentiality.

## Data Availability

No datasets were generated or analysed during the current study.
